# Partial Endovascular Embolization of a Cerebral Arteriovenous Malformation in a Patient With Seizures Caused by a Steal Phenomenon: A Case Analysis

**DOI:** 10.7759/cureus.60499

**Published:** 2024-05-17

**Authors:** Kiril Ivanov, Stanimir Atsev, Petar-Preslav Petrov, Ilko Ilyov, Plamen Penchev

**Affiliations:** 1 Faculty of Medicine, Medical University of Plovdiv, Plovdiv, BGR; 2 Cardiac Surgery Clinic, Passau Clinic, Passau, DEU; 3 Department of Anatomy, Histology and Embryology, Medical University of Plovdiv, Plovdiv, BGR

**Keywords:** steal phenomenon, clinical case report, migraine headaches, photosensitive seizures, endovascular embolization, cerebral arteriovenous malformation

## Abstract

Cerebral arteriovenous malformations (cAVMs) are developmental pathologic lesions of the blood vessels of the brain in which multiple arteries shunt blood directly into the venous drainage network. They are lesions with an unclear etiology and, if left untreated, can bear significant risks of complications such as migraines, seizures, neurological deficits, and intracranial hemorrhages. The diagnosis is based on several imaging methods, with angiography being the primary method. Treatment modalities include microsurgery, radiosurgery, embolization with the intent of obliteration, and various multidisciplinary approaches. We aim to introduce the case of an adult female patient with symptomatic cAVM who underwent partial endovascular embolization of the lesion and evaluate her recovery and the overall reliability of her treatment modality. A 22-year-old female patient has presented to the Neurosurgery Clinic with clinical manifestations with photosensitive seizures, migraines, and a history of sleep disturbances persisting for a period of one year. An appointed MRI and angiography revealed the presence of a glomerular cAVM of the anterior parietal branch of the middle cerebral artery located within the intraparietal sulcus of the left cerebral hemisphere (Spetzler-Martin grade 2). The venous drainage of the malformation led to a loss of nutrients in the surrounding brain parenchyma (a steal phenomenon), causing the seizures. The patient successfully underwent transarterial endovascular embolization with Onyx, which proved to be partial on a postoperative angiography, and refused further embolization procedures. There were no postoperative complications to be mentioned. The patient reported no seizures or sleep disturbances at the 12-month follow-up, with sporadic weak headaches remaining. cAVMs remain a pathology with significant morbidity and mortality when undiagnosed. Symptomatic cAVMs leading to a steal phenomenon and seizures can be reliably managed via endovascular embolization alone when the malformation has an appropriate angioarchitecture, location, size, and a low Spetzler-Martin score. However, further inquiry is required into the use of partial embolization in cases where further multiple-stage embolization procedures are declined and/or complete occlusion of the lesion is unfeasible. This case report emphasizes that partial endovascular embolization can be successfully utilized as a treatment modality for the symptoms caused by a steal phenomenon of the venous drainage of a cAVM, such as seizure disorders and migraines, in the rare instance when multiple-stage embolization is declined by the patient and occlusion of the lesion remains subtotal.

## Introduction

Cerebral arteriovenous malformations (cAVMs) are congenital vascular lesions of the brain or meninges with low prevalence (0.1% in adult populations), consisting of a tangle (nidus) of pathologically developed blood vessels and a venous drainage system [1-3]. While asymptomatic in the majority of cases, changes in the structure of the malformation can lead to clinical manifestations such as intracranial hemorrhages, headaches, seizures, and neurological deficits [2,3]. Loss of nutrients in the surrounding parenchyma is also observed (a steal phenomenon) [3]. The diagnosis of the lesion is based on the use of CT scanning, MRI, MR angiography, or angiography [1,4,5]. The management of the lesion consists of several treatment modalities, such as observational (conservative) therapy, microsurgery, stereotactic radiosurgery, and endovascular embolization [1,2,5-7].

Endovascular embolization can be applied as a palliative or curative treatment modality in cases with small nidus size and a low Spetzler-Martin (S-M) score where a steal phenomenon causing seizure disorders is the main symptom, with most complications (such as neurological deficits) associated with the procedure resolving in the long term [4,7-9]. Partial embolization for the treatment of AVMs (intentional as well as in cases where further procedures are declined) can be utilized in patients with seizure disorders caused by a steal phenomenon and particular structural features of the malformation, but it is not well-researched and is associated with increased hemorrhage risk [2,8,10,11].

This report aims to present the case of a 22-year-old female patient with an extensive history of seizures and sleep disturbances caused by a steal phenomenon of a cAVM in her parietal lobe. The patient underwent partial endovascular embolization of the lesion. Additionally, this report analyzes the benefits and long-term outcomes of her treatment modality.

## Case presentation

**Figure 1:**
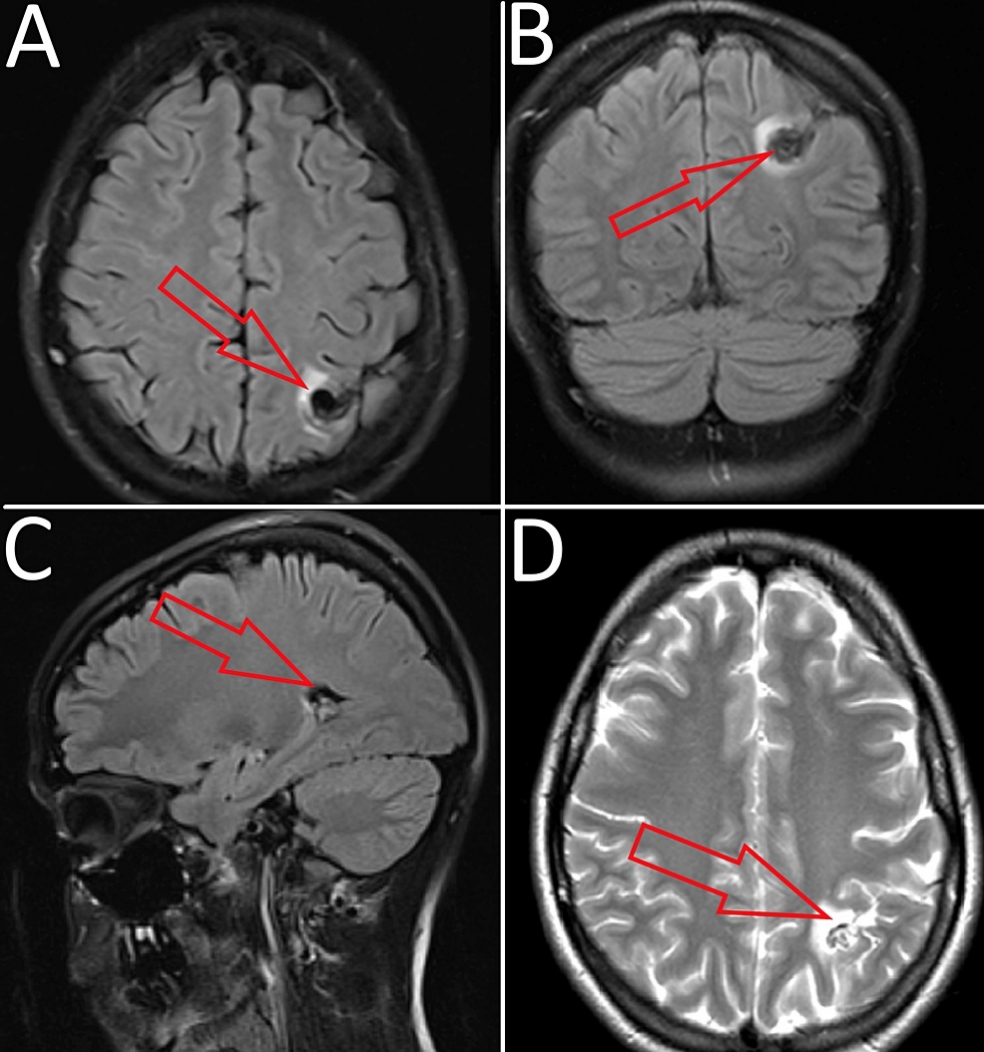
Preoperative MRI showing the location of the cAVM within the left intraparietal sulcus (A) Т1-weighted image from the axial plane; (B) T1-weighted image from the coronal plane; (C) T1-weighted image from the sagittal plane; (D) T2-weighted image from the axial plane cAVM: cerebral arteriovenous malformation

A 22-year-old female has presented to the Neurosurgery Clinic with clinical manifestations of severe hemicranial headaches akin to a common migraine (without an aura). She had a history of sporadic non-epileptic tonic-clonic seizures, during which she exhibited photophobia, along with long-lasting sleep disturbances. The manifestations persisted for a period of one year. The hemicranial headaches had a throbbing, intense nature and were described as sharp in some instances. The patient had previously treated the symptoms with over-the-counter non-steroidal anti-inflammatory drugs (NSAIDs) such as aspirin (400-600 mg orally when the migraines occurred) and ibuprofen (400 mg orally when the migraines occurred), with some success in alleviating the headaches. After a particularly severe seizure, the patient decided on a consultation with a neurosurgeon. An appointed MRI revealed the presence of a glomerular cAVM in the region of the left intraparietal sulcus (Figure 1).

**Figure 2:**
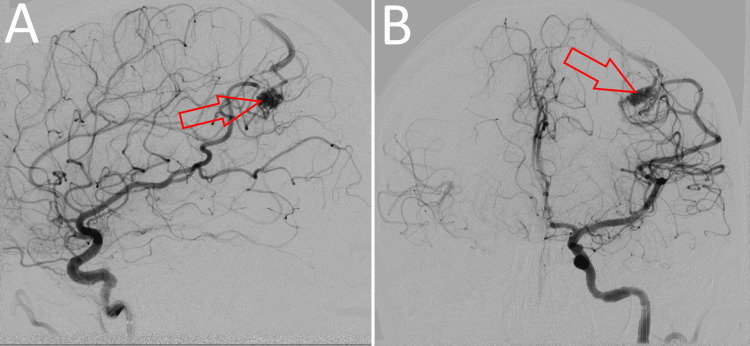
Preoperative DSA, showing the cAVM with its feeders, the main of which being the anterior parietal branch of the middle cerebral artery (A) Middle cerebral artery: lateral view; (B) middle cerebral artery: frontal view DSA: digital subtraction angiography; cAVM: cerebral arteriovenous malformation

The lesion was then imaged via a digital subtraction angiography (DSA), which revealed that the malformation was being fed by the anterior parietal branch of the middle cerebral artery (MCA) (Figure 2). A single dilated vein was draining it. Its S-M grade was determined to be 2 based on the small nidus size (2 cm) and its location in the eloquent cortex. The etiology for the seizures and sleep disturbances experienced by the patient was determined to be a loss of nutrients in the parietal lobe (a steal phenomenon) caused by the excess blood flow into the venous drainage of the nidus.

**Figure 3:**
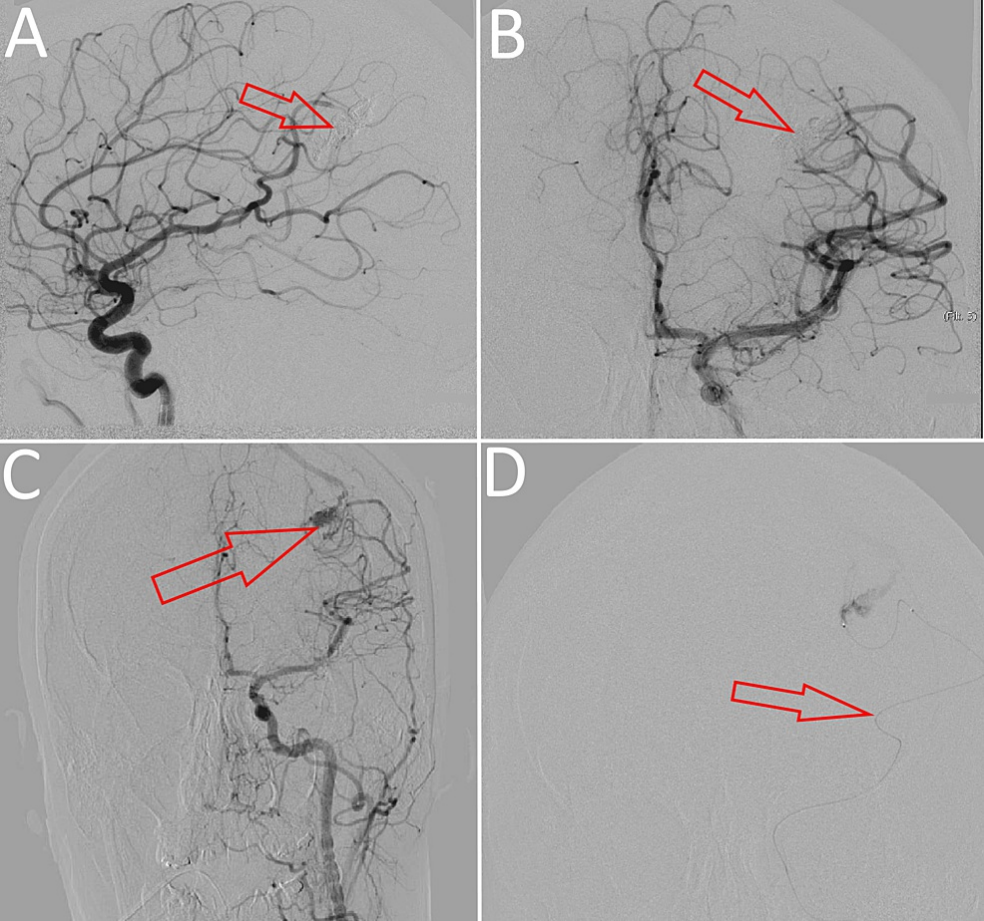
Postoperative DSA, showing subtotal occlusion of the nidus of the cAVM and obliteration of some of its arterial feeders (A) Middle cerebral artery: lateral view; (B) middle cerebral artery: frontal view; (C) internal carotid artery: frontal view showing the remaining part of the nidus; (D) superselective contrast injection in a left-side arterial feeder DSA: digital subtraction angiography; cAVM: cerebral arteriovenous malformation

Endovascular embolization with Onyx via a transarterial approach through the femoral artery was chosen as the treatment modality for the patient due to the smaller nidus size and the limited number of feeder vessels. The intervention was successful, with no intraoperative complications to be mentioned. However, a postoperative angiography revealed only a partial occlusion of the lesion (Figure 3), which was deemed significant enough to reduce the blood flow into the nidus and alleviate the symptoms of the steal phenomenon. The patient refused further treatment with the intent of complete obliteration of the malformation. The follow-up consultations revealed no postoperative complications. At the 12-month follow-up, the majority of the patient’s preoperative clinical manifestations were not present, although she complained of some sporadic headaches, which were described as less severe in nature and much rarer than the initial migraines.

EEG was not conducted in this particular case due to multiple considerations. At first, the patient experienced seizures that occurred irregularly and were not related to epilepsy, indicating a potential non-convulsive seizure disease or another underlying cause of the seizures. Furthermore, the patient displayed evident clinical indications of photophobia during the episodes, a symptom that is not frequently related to epileptic seizures but rather indicative of migraines. It was decided to go ahead with transarterial endovascular embolization based on the findings of imaging tests (MRI and DSA) and a clinical evaluation that showed the patient had a glomerular cAVM and that it was linked to their symptoms.

Prior to presenting at the clinic, the patient did not have an established prescription for anti-epileptic drugs. The choice to abstain from anti-epileptic treatment was made after consulting with the neurosurgery team, taking into account the rare occurrence of the seizures and the lack of recorded epileptic activity during clinical assessment. Furthermore, the surgical team considered the patient's tendency toward pursuing interventional treatments rather than relying on pharmaceutical methods.

## Discussion

AVMs are a developmental vascular pathology consisting of a central conglomerate of tangled blood vessels with no capillary bed, in which arteries shunt directly into the veins, forming fistulae [1]. The prevalence of cAVMs, in particular, is 0.1% in adult populations [2]. They are believed to be the result of genetic mutations as well as sporadic defects in the process of vascular morphogenesis [3]. Structural changes are frequently observed in the nidus and its venous draining system, including vessel dilatation, arterial smooth muscle hyperplasia, venous hypertension, and a “steal phenomenon,” in which brain parenchyma is deprived of oxygen and nutrients [1,2,4].

These changes can be asymptomatic or (most commonly in the age group between 20 and 50) can lead to clinical manifestations such as intracranial hemorrhages (38%-71% of symptomatic cases), seizures (18%-40%), headaches (5%-14%), and focal neurological deficits (1%-40%) [1,4]. CAVMs are designated as either parenchymal, dural, or both, depending on the location of the lesion, with the parenchymal variation further subdivided into pial, subcortical, paraventricular, or combined [4].

The diagnosis is based on the use of several imaging methods, among which contrast CT scanning and MRI (particularly weighted MRI), with angiography remaining the gold standard for the precise assessment of the nidus configuration and the scope of the venous drainage network [1,5]. In recent studies, DSA, in particular, has been recommended as a highly reliable imaging modality [5]. Differential diagnoses of the lesion include vein of Galen aneurysmal malformation, carotid/vertebral artery dissection, migraine, cerebral venous thrombosis, and various cavernous sinus and dissection syndromes [1].

For the purpose of planning out an appropriate treatment strategy, cAVMs are graded using the S-M grading system, which takes into consideration the nidus size, location of the nidus adjacent to the eloquent brain, and patterns of venous drainage in a system with grades from 1 to 5 (Table 1) [5,6]. Based on the nidus size, up to three points can be added to the grade (below 3 cm = 1 point, between 3 and 6 cm = 2, above 6 cm = 3), the location within the eloquent cortex (sensory, motor, visual, or language areas of the cortex) adds another point, and the presence of deep venous drainage can add one point; based on this system, malformations with a low grade are generally small, superficial, and non-eloquent, while those with a high grade are large in size, with deep venous drainage, and eloquent, with a special sixth grade being deemed inoperable lesions [1,5,6,12]. Studies demonstrate that a low S-M grade of a cAVM (S-M = 1 or 2) is associated with a lack of postoperative complications and neurological deficits, while those with a high grade (S-M = 4 or 5) have a significantly worse prognosis and a notable mortality risk when a surgical approach is chosen [6]. This can be observed in the case presented by us, in which a malformation with an S-M grade of 2 (nidus size: 2 cm, eloquent location, superficial drainage) underwent a smooth recovery period with no observable neurological deficits or significant clinical complications in both the short and long term.

**Table 1:** The Spetzler-Martin AVM grading system AVM: arteriovenous malformation

Spetzler-Martin AVM Grading System	Points
Size	
0-3 cm	1
3.1-6 cm	2
>6 cm	3
Location	
Non-eloquent	0
Eloquent	1
Venous drainage	
Only superficial veins	0
Deep venous drainage	1

Treatment modalities for cAVMs include conservative therapy (observation) in asymptomatic cases, alongside microsurgical excision, stereotactic radiosurgery, and endovascular embolization (with either preoperative or curative intent) in symptomatic patients [1,2,5,7]. In the clinical literature, endovascular embolization is more widely emphasized as a tool for achieving complete or partial occlusion of the arterial feeders to reduce flow into the AVM prior to its removal, and thus achieve a delay in the occurrence of an intracranial hemorrhage and also lessen the occurrence of seizures [4,9]. It has also been utilized as a palliative modality, as it can reduce the flow of blood into the venous drainage system and alleviate some of the symptoms of the steal phenomenon affecting the brain parenchyma [4].

Opinions on the efficacy and reliability of endovascular embolization as a primary curative treatment option vary. Wu et al. conclude that it is an appropriate choice in a few select cases with a deep location of the lesion, small nidus size, and a low S-M score (grade 2 or 3), although there is a notable complication risk, most significantly of vessel perforation/venous compromise (at a rate of 9.7%) and temporary neurological deficits (5.5%) [7]. The malformation of our patient covers all of the listed criteria except deep location and has no postoperative complications to be reported. Other studies note that the long-term postoperative outcomes of cAVM embolization are generally favorable, especially in patients with a nidus size smaller than 3 cm in diameter, with most neurological compromise resolving and the overall significant morbidity/mortality rate being 0.8% [8]. Such a favorable long-term outcome is demonstrated in the case we presented.

The endovascular embolization of cAVM can be performed via a transarterial or transvenous approach, with transarterial embolization being the more widely utilized method in curative as well as preoperative treatment strategies [8,9]. The transarterial approach was chosen for our patient as well (in this case, through the femoral artery) due to the presence of a well-navigable arterial feeder, namely, the anterior parietal branch of MCA. The transvenous approach is usually not preferred and, therefore, not as well-researched in the clinical literature. Chen et al. report that it can be chosen as a treatment option in a select subset of cAVMs in which there are indications such as a small nidus, deep location, obstruction of arterial access/lack of navigable arterial feeders, and the presence of a single draining vein; however, the same study emphasizes that the risks and complication rates of the intervention are not properly defined due to the limited use of the modality [9].

In most cases, when performing an endovascular embolization of a cAVM, the goal is complete occlusion of the lesion, which can necessitate multiple-stage embolization procedures, as single-stage complete obliteration is achieved in only 33% of interventions [8]. The reports on the role of partial embolization are contradictory. Ajiboye et al. conclude that only total obliteration of the lesion has clinical significance for reducing the risk of intracranial hemorrhage to zero, while Bruno et al. report that partial embolization can be a beneficial treatment modality in patients with unruptured cAVMs experiencing neurological deficits and seizures due to the occurrence of a steal phenomenon [2,8]. While further research into the conclusions drawn by Bruno et al. is needed, the case presented by us demonstrates that partial endovascular embolization can successfully resolve the seizures experienced by a patient with cAVM.

Krings et al. outline a treatment strategy utilizing partial endovascular embolization for the treatment of pial cAVMs with intranidal aneurysms, venous stenosis, and appropriate angioarchitecture [10]. We were unable to find studies regarding the postoperative outcomes and potential complications for patients who underwent partial curative embolization. However, a single study by Lv et al. reports that patients with a previous partial embolization had a higher risk of intracranial hemorrhage at initial presentation when compared with untreated individuals (3.8% versus 2.5% annually) [11]. We have not found any documented case reports regarding the outcomes and recovery of patients who refused multiple-stage embolization with the intent of total occlusion of their cAVM in favor of partial single-stage embolization.

## Conclusions

CAVMs, although a rare vascular abnormality, bear clinical significance due to the risk of severe clinical manifestations. Endovascular embolization with the intent to cure can be an appropriate and effective treatment modality for cAVMs, with favorable long-term outcomes in select cases. It can also be appropriated as a palliative modality.

We emphasize the need for further research into the use of partial endovascular embolization for the treatment of cAVMs, such as in patients with steal phenomenon leading to seizure disorders or with particular angioarchitecture of their lesions, as well as its potential complications in cases where multiple-stage embolization is declined or cannot be carried out.
